# LIPUS regulates the progression of knee osteoarthritis in mice through primary cilia-mediated TRPV4 channels

**DOI:** 10.1007/s10495-024-01950-9

**Published:** 2024-03-22

**Authors:** Sha Wu, Haiqi Zhou, Huixian Ling, Yuyan Sun, Ziyu Luo, ThaiNamanh Ngo, Yuanyuan Fu, Wen Wang, Ying Kong

**Affiliations:** Department of Rehabilitation, The Second Xiangya Hospital of Central South University, Changsha, China

**Keywords:** Low-intensity pulsed ultrasound, Primary cilia, Transient receptor potential channel 4, Chondrocyte apoptosis, Endoplasmic reticulum stress

## Abstract

Osteoarthritis (OA) is a common disease in middle-aged and elderly people. An imbalance in calcium ion homeostasis will contribute to chondrocyte apoptosis and ultimately lead to the progression of OA. Transient receptor potential channel 4 (TRPV4) is involved in the regulation of intracellular calcium homeostasis. TRPV4 is expressed in primary cilia, which can sense mechanical stimuli from outside the cell, and its abnormal expression is closely related to the development of OA. Low-intensity pulsed ultrasound (LIPUS) can alleviate chondrocyte apoptosis while the exact mechanism is unclear. In this project, with the aim of revealing the mechanism of action of LIPUS, we proposed to use OA chondrocytes and animal models, LIPUS intervention, inhibition of primary cilia, use TRPV4 inhibitors or TRPV4 agonist, and use Immunofluorescence (IF), Immunohistochemistry (IHC), Western Blot (WB), Quantitative Real-time PCR (QP) to detect the expression of cartilage synthetic matrix and endoplasmic reticulum stress markers. The results revealed that LIPUS altered primary cilia expression, promoted synthetic matrix metabolism in articular chondrocytes and was associated with primary cilia. In addition, LIPUS exerted a active effect on OA by activating TRPV4, inducing calcium inward flow, and facilitating the entry of NF-κB into the nucleus to regulate synthetic matrix gene transcription. Inhibition of TRPV4 altered primary cilia expression in response to LIPUS stimulation, and knockdown of primary cilia similarly inhibited TRPV4 function. These results suggest that LIPUS mediates TRPV4 channels through primary cilia to regulate the process of knee osteoarthritis in mice.

## Introduction

Osteoarthritis (OA) is the most common chronic degenerative bone and joint disease in the middle-aged and elderly population. It cumulatively affects 500 million people worldwide, and its main manifestation is pain, which seriously affects patients' life quality [1]. Apoptosis of chondrocytes, as the only cell type in cartilage tissue, will eventually lead to OA. [2] Endoplasmic reticulum stress (ERS) is one of the main pathways leading to chondrocyte apoptosis. [3–5] The endoplasmic reticulum is the calcium pool in chondrocytes, and together with the plasma membrane this transporter channel regulates the intracellular calcium ion concentration. Whether endoplasmic reticulum Ca2 + is imbalanced and intracellular Ca2 + concentration, both play important roles in ERS-mediated apoptosis in chondrocytes [6, 7]. Upon physical stimulation, Ca2 + signaling is involved in the regulation of mRNA expression levels of intracellular ACAN, type II collagen (COL II), and sGAG, and has an important role in transducing mechanical stimuli for the maintenance of cartilage ECM [8]. These findings corroborate the involvement of Ca2 + signaling in the regulation of cartilage degenerative processes. The regulation of intracellular calcium concentration in chondrocytes is mediated through plasma membrane calcium transport channels in addition to the release of calcium from the endoplasmic reticulum, which serves as an intracellular calcium pool. Transient receptor potential vanilloid 4 (TRPV4), a mechanosensory molecular protein in the chondrocyte membrane, regulates intracellular calcium homeostasis in chondrocytes in response to a variety of physicochemical stimuli (temperature, low osmolarity, mechanical stimuli, etc.). It plays a key role in chondrocyte matrix metabolism [9, 10]. TRPV4 is expressed both in the primary cilia and plasma membrane of cells [11, 12].

Primary cilia are present in most mammalian cell types [13] and are a specialized cellular structure with microtubules acting as axoneme protruding from the cell surface, which mediate mechanical and chemical signals. Expression of primary cilia exists in chondrocytes [14–17]. Chang et al. [14] found that primary cilia were absent from chondrocytes after conditional knockout of intraflagellar transport (IFT) 88 in mice, while articular cartilage catabolic matrix including matrix metalloproteinase 13, platelet-responsive protein deintegrin, metallopeptidase 5, collagen type X, and Runt-related transcription factor 2, as well as a significant decrease in cartilage stiffness, confirm the relevance and role of primary cilia in cartilage development and cartilage-associated diseases [18–22].

Low-intensity pulsed ultrasound (LIPUS) is a non-invasive mechanical energy delivered through the skin. Due to its mechanical, cavitation, thermal, and chemical effects, it can generate high-frequency pressure waves within biological organs and is widely used in clinical settings. It has been demonstrated that LIPUS significantly promotes the regeneration of bone and cartilage tissues with high mechanical sensitivity [23–25]. The purpose of our study is to demonstrate that LIPUS, as a mechanical stress wave, regulates the ERS of chondrocytes through mechanically sensitive primary cilia and calcium transport channel TRPV4, affecting chondrocyte apoptosis while regulating chondrocyte synthesis matrix metabolism, ultimately altering the progression of osteoarthritis.

## Materials and methods

### Animals

Specific pathogen-free grade C57BL/6 J mice, 1 week old and 6 weeks old, were purchased from Hunan Slaughter Kingda Laboratory Animal Co. Animal license number: SYXK (Hunan) 2017–0002. *Ift88*^*fl/fl*^ mice and mice expressing Cre recombinase under the control of COLII promoter (*COL II α Cre*) were obtained from Shanghai Model Organism. Experimental crosses were set up as *COL II αCre;Ift88*^*fl/wt*^* X Ift88*^*fl/fl*^. *COL II α Cre;Ift88*^*fl/wt*^ or *Ift88*^*fl/fl*^ littermates were used as controls to compare with *COL II αCre*;*Ift88*^*fl/fl*^ mutants. *COL II αCre*;*Ift88*^*fl/fl*^ mice were induced with tamoxifen (T5648, Sigma‒Aldrich, USA). Tamoxifen was administered via intraperitoneal injection once every 24 h for 5 consecutive days, and a 7-day waiting period was needed between the final injection and surgical operation. All experimental animals applied in this experiment were kept and experimented in the barrier system of the Experimental Animal Center of the Second Xiangya Hospital of Central South University. This experiment was approved by the Animal Ethics Committee of the Second Xiangya Hospital of Central South University (Approval No. 2020186), and the animal research experiments were conducted in accordance with animal ethics and welfare guidelines.

### Surgery

The ACLT + PM surgery was performed on the right knee joints, as described in our previous study. 20 mice were then randomly divided into two groups, including the ACLT + PM group and the ACLT + PM + LIPUS group (n = 10, each group). Additionally, we used the left knee joint (healthy knee joint) as the control group. To avoid wound infection, we began treatment with LIPUS (30 mW/cm2, 20 min/day) (Smith & Nephew Exogen 4000, USA) 4 weeks after ACLT + PM surgery.

### Immunohistochemistry (IHC)

The knee joint sections were dewaxed with xylene and subjected to heat-mediated antigen retrieval before the sections were sealed with goat serum. After incubation with the primary antibody overnight, the secondary antibody and horseradish peroxidase-labeled streptavidin biotin were added. Then, sections were dyed with methyl green. Primary antibodies: COLII (1:1000 dilution; ARG20787, Arigobio), ACAN (1:400 dilution; 13880-1-ap, Proteintech), BIP (1:200 dilution; 3177s, CST), β-catenin (1:100 dilution; 9562S, CST).

### TUNEL

The knee joint sections are fixed, permeabilized, and then incubated with the TUNEL reaction mixture (11,684,817,910, Roche) to label apoptotic cells. After washing and counterstaining, the sections are mounted and observed under an optical microscope for apoptotic cell detection.

### Isolation of chondrocytes and in vitro model of OA-like chondrocytes

Mouse primary chondrocytes were harvested from knee articular cartilage of 1 week old C57BL/6 J mice. The cartilage tissues were diced into and digested with 0.25% trypsin (Gibco, USA) for 30 min, then with 0.1% collagenase II (Gibco, USA) for 4 h at 37 °C. The cells were then passed through the 40 μm cell strainer to disperse the cells into single cells before seeding at a density of 2 × 104 cells/cm2. The culture was maintained in DMEMF12 supplemented with 10% FBS and 1% penicillin–streptomycin under a humidified atmosphere of 5% CO_2_ at 37 °C. The medium was changed every 3 days. For all experiments described, the chondrocytes in monolayer culture were used between passages 1–3. For the vitro model of OA, chondrocytes were induced to express an OA-like phenotype by IL-1β treatment (PeproTech, USA). IL-1β (10 ng/ ml) was added to the chondrocyte and cultured for 48 h.

Human primary chondrocytes were harvested from patients with knee osteoarthritis knee arthroplasty and patients with osteosarcoma resection of the knee. Cells were cultured in the same manner as described above.

### RNA extraction and real time RT-PCR analysis

The chondrocytes were resuspended in Trizol reagent (Invitrogen). RNA was extracted according to the manufacturer’s protocol, and treated with DNase I before real time reverse transcription polymerase chain reaction (RT-PCR) analysis. All the primers for real time RT-PCR were designed so that the product would cross intron boundaries.

### Western blotting

Chondrocytes were collected by passing the digesting medium through 40 mm cell strainer and centrifuge at 500 g for 5 min. Cell pellet was resuspended in Radioimmunoprecipitation assay buffer (RIPA; 50 mM Tris, 150 mM NaCl, 0.1% sodium dodecyl sulfate, 0.5% sodium deoxycholate, 1% Triton X 100) containing protease and phosphatase inhibitors, and sonicated for 10s. After centrifuge at 12,000*g*, 4 °C for 10 min, the supernatant was collected and 100 mg protein from each sample was used for western blotting. Goat polyclonal anti-NF-κB antibody (Abcam) was used to detect both NF-κB full length and processed repressor forms.

### Immunofluorescence(IF)

Chondrocytes were fixed for 10 min in 4% paraformaldehyde. Then, Chondrocytes were permeabilised with 0.5% Triton X-100 and blocked with 5% goat serum. Labelling with primary antibodies was performed overnight at 4 °C. The primary antibodies used were acetylated α-tubulin (1:400 dilution; T7451, Sigma-Aldrich), Anti-COL II (1:200 dilution; CY1019, Abway), Anti-ACAN(1:100 dilution; A8536, Abclone), Anti-BIP(1:200 dilution; 3177 s, CST).Following repeated washing, samples were incubated with Alexa Fluorconjugated secondary antibodies (Life Technologies) for 1 h at room temperature.Coverslips were mounted with Antifade Mounting Medium with DAPI ( P0131, beyotime). Z-stacks were generated throughout the entire cellular profile using a z-step size of 0.5 µm and reconstructed in a maximum intensity projection for quantification of cilia length and prevalence.

### Calcium imaging

The cells were serum-starved for 12–16 h prior to the experiment. Cells were gathered from the culture, resuspended in Earles’ balanced salt solution (EBSS), and then incubated in the dark at room temperature with 5 mM Fura-2AM (Invitrogen, Carlsbad, CA, USA). After incubation, the cells were rinsed in EBSS to remove the uncombined Fura-2AM. Cells were imaged using an Olympus IX71 fluorescence microscope with a 20 × objective (Olympus, Tokyo, Japan). The emission intensities under 340 nm and 380 nm illumination were recorded every 3s. The ratio of the emission densities (F340/F380) was used to convert the fluorescence intensity of the labeled cells into an intracellular calcium concentration based on known calcium standards.

### Chromatin immunoprecipitation and quantitative PCR

Chromatin immunoprecipitation (ChIP) was performed in cells. In brief, cells in 15 cm culture dishes were cross-linked with 1% formaldehyde and quenched by glycine. Cells were then lysed and nuclei were treated with micrococcal nuclease for 15 min at 37 °C. Samples were then sonicated to disrupt nuclear membrane. After centrifugation, the supernatant was collected which contained the chromatin. Chromatin solutions were incubated with antibodies anti-NF-κB(abcam, England) and anti-normal rabbit IgG (CST, USA). And then, they were rotated at 4 °C overnight, followed by incubation at 4 °C with ChIP‐grade protein G agarose beads. Next, the beads were washed. The cross-links were reversed at 65 °C for 1.5 h, and DNA was purified and used for ChIP‐qPCR analysis.

### Statistical methods

GraphPad Prism 9.5 software was used for statistical graphing. The experimental data were expressed as mean ± standard deviation, with t-test for comparison between two groups, one-way ANOVA for comparison between multiple groups, and Tukey's method for multiple comparisons. The two-sided test level was α = 0.05.

## Result

### LIPUS regulates the synthesis of metabolic matrix and alters primary cilia expression in human articular chondrocytes

**Fig. 1:**
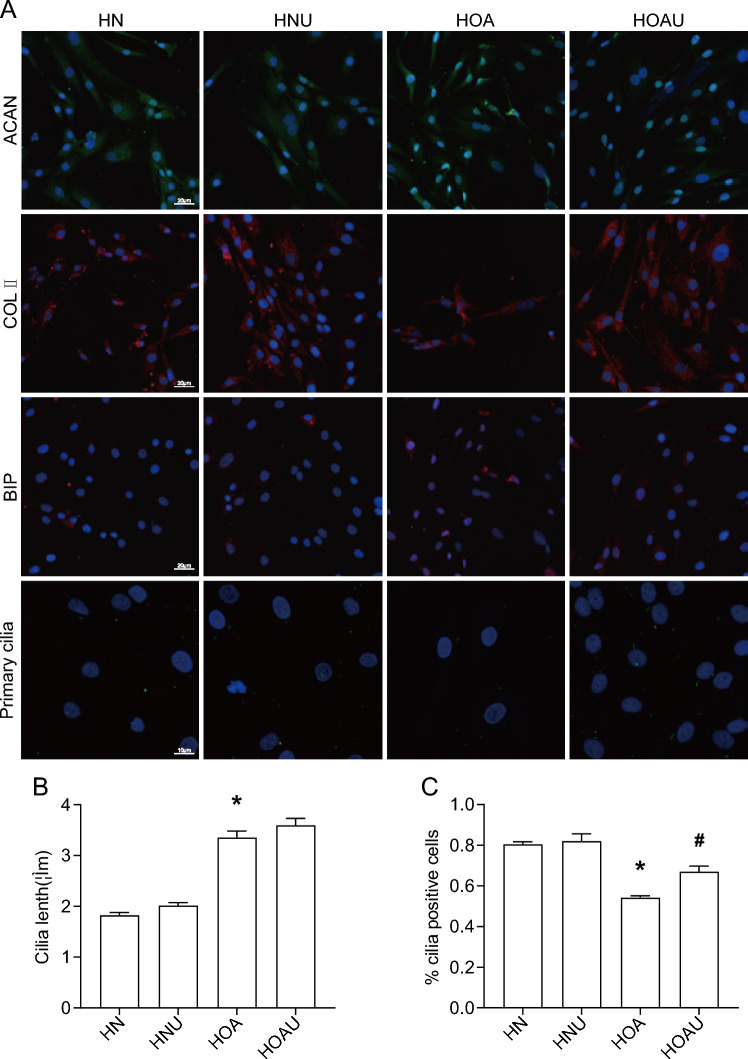
The efects of IL-1β and LIPUS in human chondrocytes. **A** Expression of ACAN, COL II, BIP and Primary cilia; **B** cilia lenth(um); **C** %cilia positive cells; Data are presented as mean ± SEM; *p < 0.05 compared to HN; ^#^p < 0.05 compared to HOA, (n = 100). *ACAN* aggrecan, *COL II* collagen II, *BIP* Immunoglobulin heavy chain binding protein, *HN* human + normal chondrocyte, *HNU* human + normal + Low intensity pulsed ultrasound chondrocyte, *HOA* human + osteoarthritis chondrocyte, *HOAU* human + osteoarthritis + Low intensity pulsed ultrasound chondrocyte

LIPUS has been well documented to play an important role in protecting the knee from Cartilaginous degradation. To investigate the anti-cartilaginous degradation capacity of LIPUS in human chondrocytes, normal and OA human chondrocytes were cultured and subjected to ultrasound intervention. Expression levels of cartilage synthesis matrix: COLII, ACAN, ERS marker BIP were detected by IF. As shown in Fig. 1A, ACAN expression was up-regulated, COLII expression was significantly decreased, and BIP expression was up-regulated in HOA chondrocyte group compared to HN group, which was reversed by LIPUS intervention.A consistent trend was also detected in the QP results, which can be proved by the trend of changing RNA levels of COLII, ACAN and BIP. These results suggest that LIPUS regulates synthetic matrix metabolism in human articular chondrocytes.

To illustrate the mechanism of mechanical action of LIPUS, we examined primary cilia,a organelles that can sense mechanical stimuli. As shown in Fig. 1A, IF showed that the cilia length was 1.83um and the incidence was 80% in the HN group, whereas in the HOA group, the cilia length was increased and the cilia incidence was decreased compared to the HN group, and in the HOAU group, the cilia length was increased and the cilia incidence was up-regulated compared to the HOA group, and there was no difference in the level of the cilium length and the level of the cilium incidence compared to the HN group. These results suggest that LIPUS alters the expression of primary cilia in human OA chondrocytes.

### LIPUS regulates synthetic matrix expression, ERS, apoptosis, and alters primary cilia expression in mouse articular cartilage

**Fig. 2:**
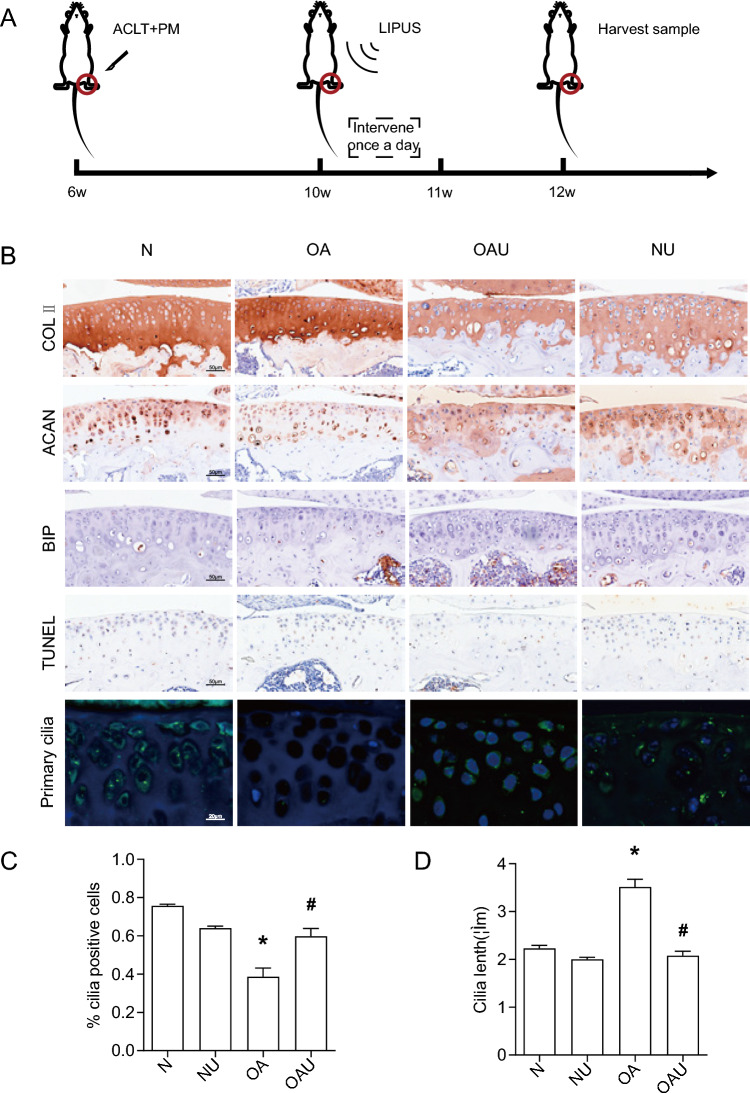
The efects of LIPUS in mouse articular cartilage. **A** Mouse OA modeling and LIPUS intervention procedure; **B** Expression of ACAN, COL II, BIP, TUNEL and Primary cilia; **C** %cilia positive cells; **D** cilia lenth(um); Data are presented as mean ± SEM; *p < 0.05 compared to N; ^#^p < 0.05 compared to OA, (n = 100). *N* sham, *NU* sham + Low intensity pulsed ultrasound, *OA* osteoarthritis, *OAU* osteoarthritis + Low intensity pulsed ultrasound

In order to verify the mechanism of action of LIPUS on cartilage, a mouse knee joint OA model was established and ultrasound intervention was performed on the knee joint (Fig. 2A). As shown in Fig. 2B, IHC results showed that COLII was uniformly distributed in knee cartilage in group N, COLII staining was uneven in OA, the degree of COLII staining in cartilage defects was significantly enhanced compared to that of group N, and the degree of COLII staining in cartilage without defects was significantly reduced compared to that of group N. In the OAU group, COLII was uniformly distributed, and the degree of staining was converged to group N. In group N, the level of ACAN The expression level of ACAN in knee cartilage of group N was significantly higher than that of group OA, while ACAN in group OAU was uniformly distributed and the degree of staining was similar to that of group N. The expression level of BIP in articular cartilage of group OA was significantly higher than that of group N, and the level of BIP in articular cartilage of group OAU was significantly lower than that of group OA. This result suggested that the ERS level of articular cartilage in OA mice was higher than that in normal mice group, and the LIPUS intervention significantly down-regulated the ERS level of articular cartilage in OA mice. TUNEL staining showed that the rate of apoptosis-positive cells in articular cartilage was significantly higher in OA group than that in N group, and the rate of apoptosis-positive cells in articular cartilage in OAU group was significantly lower than that in OA group. These results suggested that compared with the normal mouse group, the level of anabolic matrix metabolism was downregulated and apoptosis level was upregulated in articular chondrocytes of OA mice, while LIPUS intervention significantly upregulated the level of anabolic matrix metabolism and downregulated the level of apoptosis in OA mice.

Figure 2B–D showed that the cilia length in group N was 2.23um and the incidence rate was 76%, while in group OA compared to group N, the cilia length was increased and the cilia incidence rate was decreased, in group OAU compared to group OA, the cilia length was decreased and the cilia incidence rate was up-regulated, and there was no difference in the level of cilia length and the level of cilia incidence rate compared to group N. This indicates that LIPUS also altered the expression of primary cilia in mouse articular cartilage.

### LIPUS regulates mouse articular cartilage synthetic matrix expression, ERS, and apoptosis through primary cilia

**Fig. 3:**
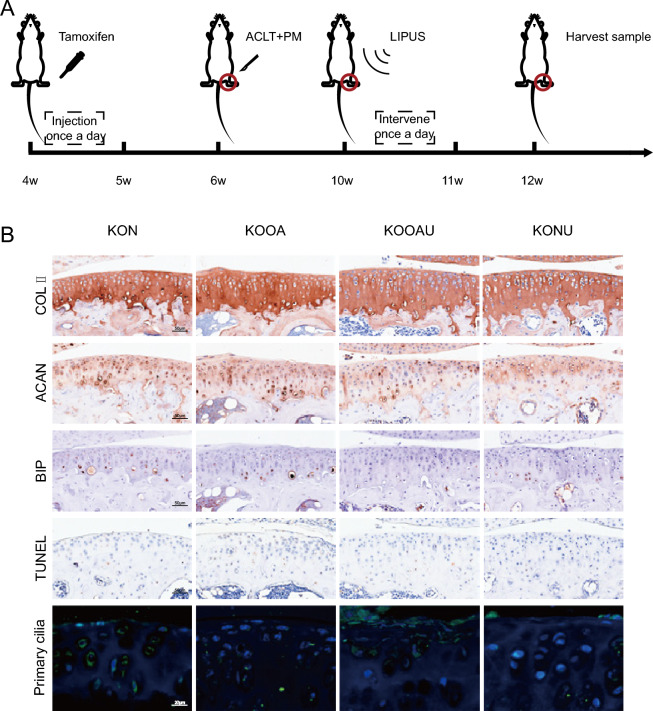
LIPUS regulates mouse articular cartilage through primary cilia. **A** *COL II Cre*;*Ift88*^*fl/fl*^ mice induced knockdown, mouse OA modeling and LIPUS intervention procedure; **B** Expression of ACAN, COL II, BIP, TUNEL and Primary cilia; *ACAN* aggrecan, *COL II* collagen II, *BIP* Immunoglobulin heavy chain binding protein, *TUNEL* TdT-mediated dUTP nick end labeling, *KO* Cartilage-Specific *IFT88* Flox mice, *N* sham, *NU* sham + Low intensity pulsed ultrasound, *OA* osteoarthritis, *OAU* osteoarthritis + Low intensity pulsed ultrasound

The above studies revealed that LIPUS altered both cartilage synthetic matrix metabolism and primary cilia expression. Therefore, we further investigated whether there was a correlation between LIPUS-induced alterations in cartilage synthetic matrix and primary cilia. IFT88 is an essential transport protein for primary cilia synthesis, so we established a conditional knockout mouse model of IFT88 and performed OA modeling and ultrasonographic interventions on the knee joints of the mice (Fig. 3A). IFT88 expression level was significantly down-regulated, indicating that IFT88 knockdown was successful. As shown in Fig. 3B, low expression of IFT88 significantly decreased the primary cilia-positive cell rate (P < 0.05), indicating that cilia were impaired in IFT88 knockout mice.

The synthetic matrix metabolism level, ERS level and apoptosis level were altered after low expression of IFT88. As shown in Fig. 3B, there was no significant difference in COLII in KON group compared to N group, no significant difference in KOOA group compared to OA group, and COLII expression in KOOAU group showed an approximate uneven distribution in OA group.There was no significant difference in ACAN in KON group compared to N group, increased in KOOA group compared to OA group, and decreased in ACAN in KOOAU group compared to OAU group. BIP of KON group had a The expression level of BIP in the KON group was significantly higher than that in the N group, and that in the KOOA group was significantly higher than that in the OA group. The expression level of BIP in the KOOAU group was significantly higher than that in the OAU group. The rate of articular cartilage apoptosis positive cells in the KON group was significantly higher than that in the N group, and that in the KOOA group was significantly higher than that in the OA group. The rate of articular cartilage apoptosis positive cells in the KOOAU group was significantly higher than that in the OAU group. These results suggest that the role of LIPUS in repairing cartilage synthesis matrix and inhibiting apoptosis may be partially inhibited by knocking out cartilage primary cilia.

### LIPUS produces a active effect on OA mouse by activating TRPV4, causing calcium inward flow

**Fig. 4:**
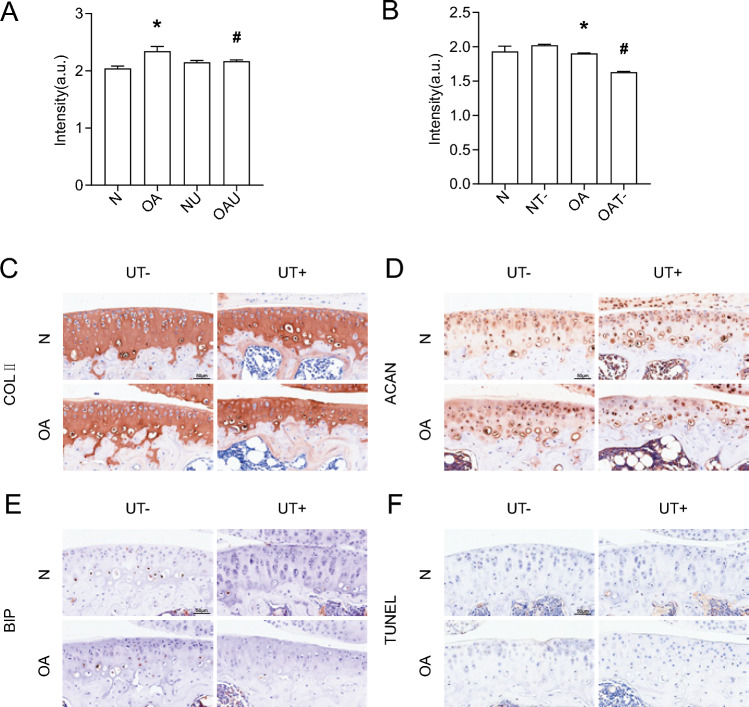
The efects of LIPUS and TRPV4 in mouse articular cartilage. **A** Calcium; **B** Calcium; **C** Expression of COL II; **D** Expression of ACAN; **E** Expression of BIP; **F** TUNEL; *p < 0.05 compared to N; ^#^p < 0.05 compared to OA, (n = 3). *N* normal chondrocyte, *NU* norma + Low intensity pulsed ultrasound chondrocyte, *OA* osteoarthritis chondrocyte, *OAU* osteoarthritis + Low intensity pulsed ultrasound chondrocyte, *NT*- normal + GSK219 chondrocyte, *OAT*- osteoarthritis + GSK219 chondrocyte, *N* sham, *OA* osteoarthritis, *UT*- Low intensity pulsed ultrasound + GSK219, *UT* + Low intensity pulsed ultrasound + GSK101

To explore the specific mechanism by which LIPUS regulates cartilage through primary cilia, we found that there is TRPV4 expression on primary cilia, which is a mechanosensitive calcium channel. Calcium flow results showed that OA chondrocyte calcium endocytosis was increased compared to N, and LIPUS could cause a decrease in OA chondrocyte calcium endocytosis (Fig. 4A). Intervention of OA chondrocytes using GSK219, an inhibitor of TRPV4, resulted in a significant decrease in calcium in-flow, confirming that the function of TRPV4 in mediating chondrocyte calcium in-flow can be inhibited by GSK219 (Fig. 4B).

We then explored whether TRPV4 plays a role in LIPUS regulation of cartilage. TRPV4 was inhibited with GSK219, and LIPUS intervention was performed on OA mice, and the histological results showed that the expression levels of COLII and ACAN in the OAUT- group were significantly decreased compared with that of the OAU group, and the expression level of BIP and the level of apoptosis in the OAUT- group were significantly up-regulated compared with that of the OAU group (Fig. 4); GSK219 intervention and ultrasound intervention were performed on OA chondrocytes, and the QP results showed that the COLII and ACAN gene expression levels were significantly decreased in the OAUT- group compared with the OA group (Fig. 4). It confirmed that TRPV4 was indeed regulated by LIPUS.

### LIPUS-stimulated primary cilia interact with TRPV4

The above studies showed that LIPUS regulates cartilage both through primary cilia and TRPV4. Therefore, we continued to explore the correlation between primary cilia and TRPV4 under LIPUS stimulation.

**Fig. 5:**
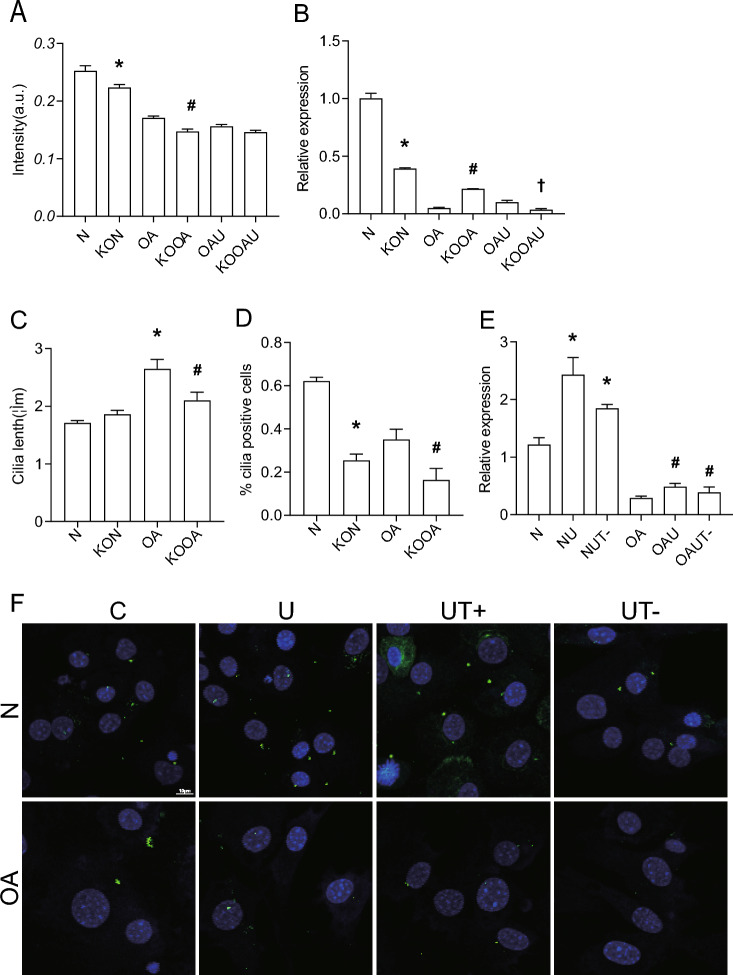
LIPUS-stimulated primary cilia interact with TRPV4. **A** Calcium; **B** Expression of IFT88; **C** cilia lenth(um); **D** %cilia positive cells; **E** Expression of IFT88; **F** Expression of Primary cilia; Data are presented as mean ± SEM; *p < 0.05 compared to N; ^#^p < 0.05 compared to OA, †p < 0.05 compared to OAU,(n = 100). *N* normal chondrocyte, *OA* osteoarthritis chondrocyte, *OAU* osteoarthritis + Low intensity pulsed ultrasound chondrocyte, *KON* sh-IFT88 + normal chondrocyte, *KOOA* sh-IFT88 + osteoarthritis chondrocyte, *KOOAU* sh-IFT88 + osteoarthritis + Low intensity pulsed ultrasound chondrocyte, *NUT*- normal + Low intensity pulsed ultrasound + GSK219 chondrocyte, *OAUT*- osteoarthritis + Low intensity pulsed ultrasound + GSK219 chondrocyte, *C* control, *U* Low intensity pulsed ultrasound, *UT*- Low intensity pulsed ultrasound + GSK219, *UT* + Low intensity pulsed ultrasound + GSK101

Lentiviral transfection was used to knock down ift88 expression, and QP results showed that lentiviral knockdown efficiency reached 50% (Fig. 5B), and WB results illustrated that knockdown of IFT88 resulted in a significant decrease in primary cilia expression.IF results showed that primary cilia length was significantly shorter in the KOOA group compared to the OA group (Fig. 5C), and cilia incidence was significantly decreased in the KON group compared to the N group (Fig. 5D), and calcium ion results showed a decrease in the KON group compared to the N group, and a decrease in the KOOA group compared to the OA group, and knockdown of IFT88 would inhibit calcium ion influx in normal chondrocytes and OA chondrocytes.

Intervention of chondrocytes using GSK219 to change TRPV4 function, followed by ultrasound intervention, QP results showed that the RNA level of IFT88 in the NU group was higher than that of the N group, while the RNA level of IFT88 in the NUT- group was decreased compared to that of the NU group.The RNA level of IFT88 in the OAU group was higher than that of the OA group, while the RNA level of IFT88 in the OAUT- group was decreased compared to the OAU group. The IF results showed that the ciliogenesis was higher in the NU group than in the N group, while the ciliogenesis was decreased in the NUT- group compared with the NU group. The ciliogenesis was higher in the OAU group than in the OA group, while the ciliogenesis was decreased in the OAUT- group compared with the OAU group. The above results suggested that GSK219 inhibited the expression of primary cilia (Fig. 5E,F).

Taken together, it suggests that primary cilia interact with TRPV4 in chondrocytes under ultrasound intervention.

## Discussion

OA is an active dynamic change caused by an imbalance between repair and destruction of joint tissues, rather than a passive degenerative disease or so-called wear-and-tear disease as it is commonly described. Articular cartilage is a kind of mechanosensitive tissue. Under a variety of pathological stimuli, such as inappropriate loading, the extracellular matrix is gradually degraded, which subsequently causes an imbalance of forces in the cartilage tissue, and then leads to chondrocyte apoptosis. Chondrocytes, as the only cells existing in cartilage tissue, can feel external stimuli and respond accordingly, which is the key factor to maintain the stability of the internal and external cellular environment. The morphology and number of chondrocytes play an important role in extracellular matrix secretion and synthesis, and chondrocyte apoptosis will eventually lead to OA [25]. In this study, OA triggered or mimicked by IL-1β-induced inflammatory response was investigated [26, 27]. LIPUS is a non-invasive therapeutic method delivered percutaneously, characterized by lower energy intensity as well as output in the form of pulsed waves, which can produce thermal, cavitation and mechanical effects in biological organs by modulating gene expression, cellular signaling, influencing enzyme activity, cell proliferation and differentiation, cytokine secretion, angiogenesis, and stem cell differentiation to exert anti-inflammatory and analgesic effects [28]. The amplitude of ultrasound determines the intensity of the LIPUS signal [29–32]. The application of LIPUS at low intensity does not cause physiological stress, and after 10 min of action, the temperature rises by only 0.5 °C.[33, 34] The therapeutic action of LIPUS is mainly dependent on its non-thermal effect, and LIPUS has the advantages of non-invasive, inexpensive, portable, and with obvious therapeutic efficacy, which is now widely used in clinics. The earliest use of LIPUS was for clinical diagnosis, including vital organs, fetal development, and bone scaffold growth evaluation in the process of fracture healing [28]. LIPUS was first used for clinical diagnosis, including the evaluation of important organs, fetal development, and bone scab growth during fracture healing, and then it was found to have a significant effect on the regeneration of bone and cartilage tissues with high mechanical sensitivity, and then gradually applied to the treatment of bone and cartilage [35, 36]. It has been suggested that it is mainly delivered to cells and tissues in the form of fluid shear stress and mechanical stimulation through cavitation, acoustic flow and nanoscale micromotion [28]. LIPUS promotes ACAN synthesis [23], and improves the morphology and histological appearance of damaged articular cartilage in rat and rabbit OA models in vivo. [24, 25]Sang et al.[37] showed that LIPUS promotes bone and cartilage regeneration by modulating the focal adhesion kinase FAK signaling pathway to promote chondrocyte proliferation and differentiation. In the present study, we similarly observed that LIPUS can regulate the synthesis of chondrocyte extracellular matrix proteins such as COLII and ACAN.

How is LIPUS sensed by chondrocytes and involved in chondrocyte and extracellular matrix regulation? We consider primary cilia are involved in this process. Primary cilia are a specialized cellular structure with a centrosome as a substrate and protruding on the surface of the cell membrane, which are present in most dormant and differentiated mammalian cells, and are capable of sensing a variety of physical and chemical stimuli in the microenvironment and participating in the transduction of multiple signaling pathways, which enables the cells to make an adaptive response to changes in the external environment. In contrast, chondrocyte primary cilia are mechanosensitive and play an important role in sensing fluid shear stress and mechanical stimuli [38, 39]. The assembly of cilia is mainly dependent on the IFT complex, and IFT88, as an essential protein during primary cilia synthesis, carries ciliary building blocks along microtubules during cilia assembly and maintenance. In addition, conditional deletion of IFT88 has been used to study cell- or tissue-specific primary cilia function [40]. In this study, we examined the gene expression level of IFT88 using QP and the incidence of primary cilia using cellular immunofluorescence assay. We found that LIPUS intervention elevated primary cilia expression and shortened the length of primary cilia in chondrocytes after IL-1β intervention, suggesting that LIPUS can regulate the expression of primary cilia in chondrocytes. Similar to the results of the present study, Matsumoto et al. [39] found that LIPUS increased the number of primary cilia at the fracture site, stimulated the number and length of primary cilia in the osteoblast-like cell line MC3T3-E1 cells, and also stimulated the levels of cilium protein, IFT88 mRNA. Huang et al. [41] used LIPUS stimulation for 3 h to significantly reduce the incidence and length of cilia in cultured neurons, and increasing the duration and intensity of LIPUS stimulation also decreased the incidence and length of cilia. Subramanian et al. [42] showed that primary cilia were elongated and curved by low-intensity ultrasound, and these changes were reversible. Oh et al. [43] investigated high-intensity focused ultrasound and found that it decreased the levels of ciliary disassembly-related factors (AurA and HDAC9) and increased the expression of cilia assembly-related factors (KIF3A and IFT88) in subcutaneous adipose tissue. In summary, primary cilia expression in chondrocytes, osteoblasts, neurons and stem cells is regulated by mechanical stimuli such as LIPUS.

Therefore, we hypothesized that ultrasound exerts a regulatory effect on chondrocyte synthetic matrix and apoptosis through primary cilia. To further verify this speculation, chondrocytes were silenced with IFT88 to observe whether the regulation of chondrocyte anabolic matrix level and apoptosis by LIPUS would be altered, and the results showed that in mouse chondrocytes induced by IL-1β, the effects of LIPUS intervention induced by increasing anabolic matrix expression and decreasing apoptosis were inhibited by lentiviral silencing of IFT88, indicating that IFT88 may be involved in the process of regulating the protective effect of LIPUS on IL-1β-induced mouse chondrocytes. Such results suggest that primary cilia have a crucial role in the chondroprotective effects of LIPUS. Similar to our results, Xiao et al. [35] showed that LIPUS is enhanced osteogenic differentiation of Prrx1 + cells (the major population of osteochondral progenitor cells in the periosteum) mainly by promoting the expression of primary cilia. Mechanical loading regulates primary cilia elongation and inhibits cartilage inflammatory signaling through HDAC6- and IFT-dependent mechanisms [44]. LIPUS promotes regeneration of femoral defects and enhances osteogenic differentiation of Prrx1 + cells. However, these effects are significantly attenuated when primary cilia of Prrx1 + cells are knocked down [35, 45]. Knockdown of the cilia assembly-associated factor KIF3A caused disruption of primary cilia and significantly reduced the amount of bone formed in response to mechanical stimulation [46]. LIPUS or other mechanical stimuli play a role in promoting cartilage synthesis and osteogenesis, which are significantly attenuated when primary cilia are knocked down, suggesting that none of these roles can be achieved without the intermediary of primary cilia.

Chondrocytes can produce spontaneous Ca2 + signals in the resting state; changes in the structure, composition, and level of inflammatory factors in the ECM during cartilage degeneration can lead to changes in the characteristics of spontaneous Ca2 + signals in OA chondrocytes. Kawano et al. [47] studied the spontaneous Ca2 + signals of in situ chondrocytes in normal and OA degeneration regions of human knee joints and found that the Ca2 + signal characteristics of cells in various layers of normal cartilage tissue had differences and were dependent on the regulation of extracellular Ca2 + concentration; the abnormalities of the internal environment of the OA cartilage tissue may be the reason for the differences in Ca2 + signals between OA chondrocytes and normal cells. Kono et al. [48] found that mechanical stimulation of a single fine within a two-dimensional culture reaching a population of fused chondrocytes induced a water-wave-like transmission of Ca2 + signaling in adjacent chondrocytes. Upon physical stimulation, Ca2 + signaling is involved in the regulation of mRNA expression levels of intracellular ACAN, COL II, and sGAG, which have important roles in transducing mechanical stimulation for the maintenance of cartilage ECM [8, 49–51]. These corroborate the involvement of Ca2 + signaling in the regulation of cartilage degenerative processes The regulation of intracellular calcium concentration in chondrocytes is mediated through plasma membrane calcium transport channels in addition to the release of calcium from the endoplasmic reticulum, an intracellular calcium pool. TRPV4 is one of the plasma membrane calcium transport channels. TRPV4 is a mechanoreceptor protein in the chondrocyte membrane that regulates intracellular calcium homeostasis in chondrocytes in response to a variety of physicochemical stimuli (temperature, low osmolarity, mechanical stimuli, etc.). It plays a key role in chondrocyte matrix metabolism [8–10]. Mechanical stimulation was found to regulate chondrocyte metabolic responses and promote cartilage matrix synthesis by activating TRPV4 channels and upregulating intracellular Ca2 + concentration [52–55]. Blockade of TRPV4 inhibits chondrocyte anabolism and promotes catabolism expression, blocking mechanically-mediated extracellular matrix accumulation and mechanical property improvement, whereas agonist activation of TRPV4 similarly enhances chondrocyte anabolism and inhibits catabolism gene expression in the absence of mechanical loading [10, 48, 56, 57]. It is evident that TRPV4 channel-mediated signaling pathway transduction plays an important role in transmitting mechanical loads, physicochemical stimuli, and maintaining cartilage extracellular matrix and normal joint function. Scholars have found that TRPV4 is expressed in the primary cilia and plasma membrane of cells [11, 12, 58–60]. Corrigan et al. [53] found that TRPV4 is expressed in the primary cilia and plasma membrane of MSC cells, and that TRPV4 can mediate oscillatory fluid shear mechano transduction in MSCs partly through primary cilia.

## Conclusion

Chondrocytes sense changes in physicochemical and mechanical signals in the extracellular microenvironment through primary cilia, activate the TRPV4 pathway, regulate intracellular Ca2 + concentration, regulate endoplasmic reticulum stress, affect chondrocyte apoptosis, and change the expression of COL II and ACAN, the constituents of the cartilage matrix. LIPUS can stimulate the primary cilia of chondrocytes, activate the TRPV4 pathway, regulate the intracellular Ca2 + concentration, inhibit the endoplasmic reticulum stress, reduce the apoptosis of chondrocytes, increase the expression of COLII and ACAN, regulate the metabolism of the cartilage matrix, change the force of the chondrocytes in the matrix, and regulate the size and morphology of the chondrocytes, so as to form the new dynamic equilibrium between the chondrocytes and cartilage matrix, and ultimately slow down the development of arthritis.

## Data Availability

The datasets generated during and/or analysed during the current study are available from the corresponding author on reasonable request.

## Abbreviations

ACAN: Aggrecan
BIP: Immunoglobulin heavy chain binding protein in pre-B cells
BMSC: Bone mesenchymal stem cells
Col II: Collagen, type II, alpha 1
ECM: ExtraCellular Matrix
ERS: Endoplasmic reticulum stress
EXO: Exosomes
GRP78: 78-KD glucose-regulated protein
IF: Immunofluorescence
IFT88: Intraflagellar transport 88
IHC: Immunohistochemistry
IL-1β: Interleukin-1 beta
LIPUS: Low intensity pulsed ultrasound
NF-κB: N + Uclear factor kappa-B
OA: Osteoarthritis
QP: Quantitative Real-Time PCR
TRPV4: Transient receptor potential channel 4
WB: Western Blot
